# Barriers and facilitators experienced by osteopaths in implementing a biopsychosocial (BPS) framework of care when managing people with musculoskeletal pain – a mixed methods systematic review

**DOI:** 10.1186/s12913-021-06720-w

**Published:** 2021-07-15

**Authors:** Kesava Kovanur Sampath, Ben Darlow, Steve Tumilty, Warwick Shillito, Melissa Hanses, Hemakumar Devan, Oliver P. Thomson

**Affiliations:** 1 Centre for Health and Social Practice, Waikato Institute of Technology, 51, Akoranga Road, Hamilton, New Zealand; 2 Department of Applied Sciences and Social Practice, Ara Institute of Canterbury, Christchurch, New Zealand; 3 School of Public Health, University of Technology Sydney, Sydney, Australia; 4 Department of Primary Health Care and General Practice, University of Otago, Wellington, New Zealand; 5 Centre for Health, Activity, and Rehabilitation Research (CHARR), School of Physiotherapy, University of Otago, Dunedin, New Zealand; 6 Department of Health Practice, Ara Institute of Canterbury, Christchurch, New Zealand; 7 Centre for Health, Activity, and Rehabilitation Research (CHARR), School of Physiotherapy, University of Otago, Wellington, New Zealand; 8 University College of Osteopathy, London, UK

**Keywords:** Musculoskeletal pain, Osteopathy, Biopsychosocial approach, Clinical practice guidelines, Barriers and facilitators

## Abstract

**Background:**

Clinical practice guidelines commonly recommend adopting a biopsychosocial (BPS) framework by practitioners managing musculoskeletal pain. However, it remains unclear how osteopaths implement a BPS framework in the management of musculoskeletal pain. Hence, the objective of this review was to systematically appraise the literature on the current practices, barriers and facilitators experienced by osteopaths in implementing a BPS framework of care when managing people with musculoskeletal pain.

**Methods:**

The following electronic databases from January 2005 to August 2020 were searched: PubMed, CINAHL, Science Direct, Google Scholar, ProQuest Central and SCOPUS. Two independent reviewers reviewed the articles retrieved from the databases to assess for eligibility. Any studies (quantitative, qualitative and mixed methods) that investigated the use or application of the BPS approach in osteopathic practice were included in the review. The critical appraisal skills program (CASP) checklist was used to appraise the qualitative studies and the Mixed Methods Appraisal Tool (MMAT) was used to appraise quantitative or mixed methods studies. Advanced convergent meta-integration was used to synthesise data from quantitative, qualitative and mixed methods studies.

**Results:**

A total of 6 studies (two quantitative, three qualitative and one mixed methods) were included in the final review. While two key concepts (current practice and embracing a BPS approach) were generated using advanced meta-integration synthesis, two concepts (barriers and enablers) were informed from qualitative only data.

**Discussion:**

Our review finding showed that current osteopathic practice occurs within in the biomedical model of care. Although, osteopaths are aware of the theoretical underpinnings of the BPS model and identified the need to embrace it, various barriers exist that may prevent osteopaths from implementing the BPS model in clinical practice. Ongoing education and/or workshops may be necessary to enable osteopaths to implement a BPS approach.

## Background

Musculoskeletal pain conditions such as low back pain, osteoarthritis and tendinopathies have a substantial influence on quality of life [1, 2] and are leading (and growing) causes of disability internationally [3]. The economic burden (direct and in-direct costs) of musculoskeletal pain is significant due to its high prevalence and impact on employment. Musculoskeletal pain is managed in primary care by range of different health care professions including medical doctors, physiotherapists, osteopaths and chiropractors [4]. There is evidence to indicate that many practitioners adopt biomedically orientated management approaches where the focus is to diagnose and treat ‘patho-anatomical’ structures as primary causes of patients’ symptoms [5, 6]. However, a biomedical model has been shown to be inadequate for managing many musculoskeletal pain conditions where no clear pathophysiological diagnosis can be reliably made and there is a complex and person-specific interaction of different factors [7] . This approach has resulted in inappropriate usage of imaging and an overuse of costly care that delivers low beneficial value [3]. Furthermore, a biomedical approach to musculoskeletal pain fails to give primacy to key psychosocial factors such as depression, anxiety, fear avoidance, social isolation and catastrophizing that have been shown to play important roles in the development and recovery of musculoskeletal pain and disability [8–10]. Consequently, evidence-based clinical practice guidelines recommend that psychosocial factors be assessed and managed in addition to biological factors when treating patients with musculoskeletal pain [11, 12].

Osteopaths are one of the groups of primary healthcare professionals who manage musculoskeletal pain conditions via a range of conservative interventions including manual therapy, exercise therapy and self-management advice [13]. Despite evidence emphasizing the assessment of psychosocial factors in pain population, osteopaths may be inclined to have a greater orientation towards the biomedical rather than the biopsychosocial (BPS) model of care [14–17] and have a relatively low utilisation of clinical practice guidelines in osteopathic practice [18–20]. The reasons for non-adherence to clinical practice guideline recommendations include inadequate knowledge and lack of time, skills and confidence to incorporate a psychologically informed approach in people with chronic low back pain [21, 22]. Further, clinical practice guidelines often recommend active interventions (e.g. exercise) over or in combination with passive interventions (e.g. manual therapy) the treatment of musculoskeletal pain [11, 12]. This may be perceived to undermine the core hands-on manual therapy skills which contribute to osteopaths’ professional identity [23].. However, factors influencing osteopaths use of BPS approach, has not been systematically reviewed [17]. Understanding the enablers and challenges towards implementing BPS approach in osteopathic practice may help toward increased uptake and implementation of this framework for optimal patient outcomes.

### Review question(s)

- What is the usage of the BPS framework in current osteopathic practice?
- What factors enable or prevent osteopaths to implement a BPS approach into their practice?
- What types of interventions would facilitate osteopaths to implement a BPS framework into their practice?

## Methods

The protocol for this review was published [24] and registered in the International Prospective Register of Systematic Reviews (CRD42020159227) [25]. Findings are reported in accordance with the Preferred Reporting Items for Systematic reviews and Meta-Analysis (PRISMA) guidelines.

### Study selection

Studies published from January 2005 onwards from any osteopathic setting (education, private practice, hospital or multi-disciplinary clinic) were included. Further, studies were included if they investigated the use or application of the biopsychosocial approach in osteopathic practice using quantitative, qualitative and mixed methods. Relevant thesis or dissertations that met the inclusion criteria were also included. The studies had to either explicitly state investigating osteopaths’ use of the BPS approach or provide enough information within the article for the review team to deduce that it explored osteopaths’ use of the BPS model.

Studies were excluded if they were a previous review (systematic, scoping and narrative), expert opinion commentary, or were published in any language other than English.

The outcome measures of interest included any instruments or questionnaires that measured the attitudes and beliefs of osteopaths towards the BPS model (e.g. Pain Attitudes and Beliefs Scale for Physiotherapists). For qualitative studies, the outcomes of interest were key themes that explored the attitudes toward uptake of BPS model of care and/or barriers and facilitators experienced by osteopaths in implementing a BPS model of care in their clinical practice.

### Identifying relevant studies (search process)

A systematic search strategy was developed by the research team in consultation with a librarian to locate studies relevant to osteopathic practice and the biopsychosocial model. A combination of keywords such as ‘manual therapy’, ‘osteopath*’, ‘spinal manipulation’, ‘manipulation, osteopathic,’ ‘thrust’, ‘OMT’, ‘biopsychosocial’, ‘BPS model’, ‘patient centeredness’, ‘patient centred approach’, ‘facilitators’, ‘enablers’, ‘challenges’, ‘barriers’, ‘usage’ and ‘implementation’ was used for this purpose. The Boolean operators “OR” and “AND” were used to combine the search terms within and between each of the subject areas, respectively. Table 1 outlines the complete search strategy used for the time period from January 2005 to August 2020. The following electronic databases were searched: PubMed, CINAHL, Science Direct, Google Scholar, ProQuest Central and SCOPUS. A secondary search through ‘grey literature’ was also undertaken on ProQuest (Dissertations and Theses), Ethos, open grey, clinical trial registries such as ANZCTR and systematic review protocol registries such as PROSPERO. Further, forward and backward citation searches from included articles or relevant reviews was undertaken to retrieve additional articles [26]. Two reviewers (K.S.K. and M.H.) independently conducted electronic searches in the above-mentioned databases. All references were exported into separate Endnote libraries (Version X7; Thomson Reuters, New York) of the reviewers.

**Table 1:** Search strategy (used for CINAHL database)

Concept 1	Concept 2	Concept 3
1. Exp. Osteopath* 2. Exp. Manual therapy 3. Osteopathic Manipulative Treatment (OMT) 4. Spinal Manipulation 5. Thrust 6. Joint mobilization 7. Or/ 1–6	8. Exp. BPS Model 9. Biopsychosocial* 10. BPS Framework/care 11. Patient centeredness 12. Patient care 13. Patient centered approach 14. Clinical Practice Guidelines 15. Musculoskeletal Pain/therapy [Mesh] 16. Or/8–15 17. 7 AND 16	18. Usage 19. Implementation 20. Facilitator 21. Enabler 22. Barrier 23. Challenges 24. Attitudes 25. Or/18–24 26. Randomized clinical trial/ 27. Controlled clinical trial/ 28. Qualitative Study 29. Mixed Methods Study 30. or/ 26–29 31. 25 AND 30 32. 17 AND 31

**Filters:** The following filters were applied: **Year:** Jan 2005 to August 2020; **Language:** English

### Article selection

After removing the duplicates, two reviewers (K.S.K. and M.H.) independently screened the titles, abstracts, and full texts of the retrieved articles against the eligibility criteria. Any disagreement between the two authors was resolved by mutual discussion. A decision could not be reached for one article for which a third reviewer (H.D.) was consulted and his decision was considered final.

### Data extraction

Two reviewers (KSK and MH) independently extracted data from each study using a standardised template. The data extracted were (1) study aim(s); (2) study design; (3) population; (4) study findings; and (5) authors’ conclusions. Data extracted by the two reviewers were reviewed and combined through discussion and consensus; and a third reviewer (H.D.) was available to resolve disagreements (but not required). H.D. compared the extracted data against the original articles to check for errors.

### Quality assessment (including risk of bias)

The critical appraisal skills program (CASP) checklist was used to appraise the qualitative studies [27] and the Mixed Methods Appraisal Tool (MMAT) [28] was used to appraise quantitative or mixed method studies. Two reviewers (K.S.K. and M.H.) independently assessed the quality of each study. Both reviewers recorded their rationale in addition to study ratings to enable comparison. Disagreements were resolved through discussion and consensus. While a third reviewer (H.D.) was available for arbitration, there were no disagreements between K.S.K and M.H.

### Data synthesis

#### Quantitative data

Data synthesis is comprehensively described in the published protocol [24]. Briefly, meta-analysis was performed using Review Manager (RevMan 5.3) software where it was possible to pool data from two or more studies using the same outcome measure.

#### Qualitative data

We used a thematic synthesis approach to synthesise qualitative data. The qualitative data (i.e. main themes and sub-themes) from the included studies were imported to NVivo (Version 11; QSR International, Victoria, Australia) and analysed in 3-steps: (1) line-by-line coding, (2) descriptive themes, and (3) analytical themes. Two reviewers (K.S.K. and H.D.) independently coded all the included articles to derive the initial descriptive themes. An iterative approach was undertaken by both authors (K.S.K. and H.D.) moving between the raw data from the original articles to identify analytical themes emerging from the synthesis. K.S.K. and H.D. used a combination of diagrams and mind maps to discuss and debate the analytical themes. The analytical themes were then presented to two experienced qualitative researchers (O.T. and B.D.), who checked the emerging themes and provided critical inputs to reduce any unintentional bias. The themes thus derived were presented to the whole team which provided opportunities for further interpretation and refinement of the analytical findings.

The GRADE-CERQual [29] (Confidence in the Evidence from Reviews of qualitative research) approach recommended by the Cochrane and Implementation Methods Group, was used to summarise the level of confidence in synthesised qualitative findings. Overall confidence was graded as high, moderate, low and very low based on the extent to which there were concerns (no or very minor; minor; moderate and serious) on each of the four CERQual components (methodological limitations, relevance, coherence and data adequacy). K.S.K. conducted the critical appraisal process of the review findings in an Excel spread sheet (Microsoft Corp, Redmond, Washington). H.D. verified the evaluation and a final decision was based on consensus between K.S.K. and H.D.

#### Combining quantitative and qualitative data

Advanced convergent meta-integration was used to synthesise data from quantitative, qualitative and mixed method studies, a recommended synthesis approach for mixed method systematic reviews [30]. This approach involved five steps: (1) categorising data sources (2) conducting intra-method analysis-synthesis and mindful comparison (3) conducting inter-method integration (4) organizing results and assess fit, and (5) drawing final conclusions. Steps two to five were iterative and provided an overview of complex inter-relational connections from the emerging data. K.S.K led the convergent meta-integration process and the emerging concepts were subsequently presented to experienced researchers (O.T., B.D., S.T. and H.D.) for critical inputs and to reduce bias. However, there are no tools that exist to summarize level of confidence from meta-integration.

## Results

### Results of the search

**Fig. 1:**
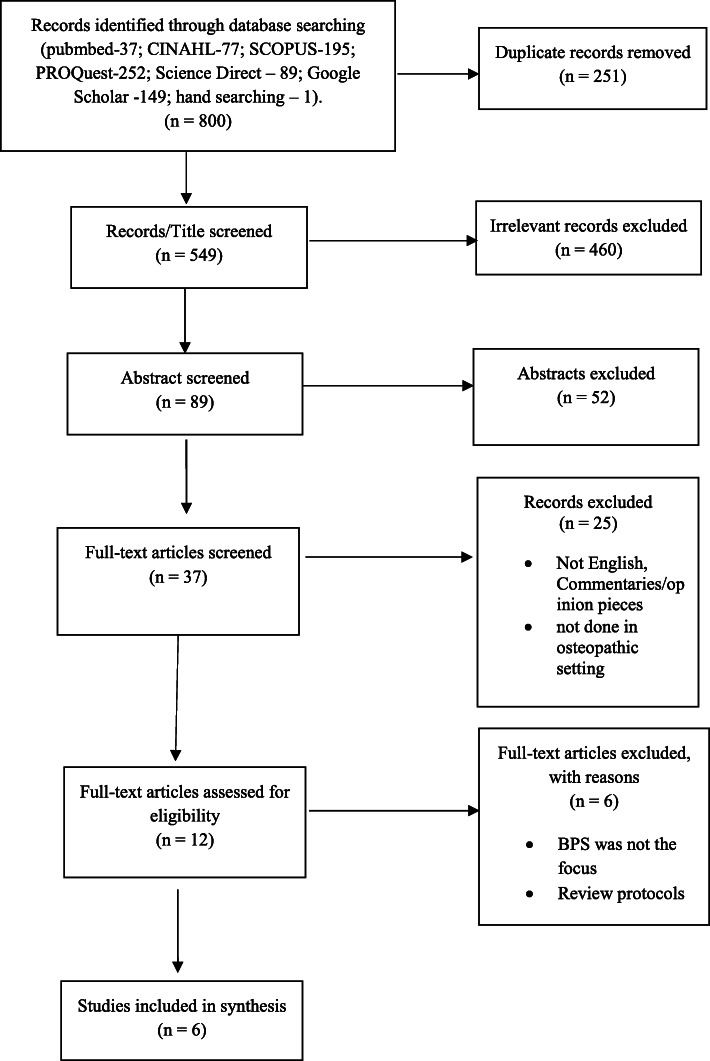
PRISMA flow diagram of included studies

Our electronic search yielded a total of 799 articles. Following the removal of duplicates, 549 articles were retained for further screening. After title, abstract, and full-text screening, six studies [14, 15, 17, 31–33] were included for final synthesis (Fig. 1). Consensus could not be achieved between K.S.K and M. H on one study for which H. D was consulted and his decision to exclude it was considered final.

### Description of included studies

A summary of the included studies is presented in Table 2. In total, 405 osteopaths contributed to the findings, of which, 368 osteopaths responded to questionnaires and 37 osteopaths were interviewed. Except for one study [32] in which the participants were final year osteopathic students, the participants in all other studies were registered osteopaths. Of the six included studies, five [14, 15, 31–33] were conducted in the United Kingdom and one study was conducted in Italy [17]. There were two quantitative studies [14, 15] that covered attitudes and beliefs; 3 qualitative studies that covered current practice, barriers and enablers for utilizing BPS approach [17, 31, 32]; and one mixed methods study that covered all these concepts [33]. The common outcome measures utilized in the studies included the Attitudes to Back Pain Scale in musculoskeletal practitioners (ABS-mp), Pain Attitudes and Beliefs Scale for Physiotherapists (PABS.PT) and Health Care Providers Pain and Impairment Relationship scale (HC-PAIRS).

**Table 2:** Characteristics of included studies

Study (Country)	Methods/Study type	Study Settings	Participants	Outcome measure/method of analysis	Main findings
Bar-Zaccay & Bailey (2018) [14] (UK)	Cross-sectional Survey	Online questionnaire (National level)	UK osteopaths *N* = 107 M = 51 F = 56	PABS-PT (BM and BPS scores) Descriptive and inferential statistics	UK osteopaths hold strong BM beliefs about pain, however, with an acceptance of the BPS approach
Macdonald et al., (2018) [15] (UK)	Cross-sectional Survey	Online questionnaire (National level)	UK osteopaths *N* = 216 M = 118 F = 98	PABS-PT HC-PAIRS Descriptive statistics	Osteopaths have skills to engage with psychosocial factors of the patients’ pain experience. However, training is required to increase their expertise in knowledge of chronic pain and its management.
Abrosimoff & Rajendran (2020) [31](UK)	Semi-structured interviews (individual)	Osteopathic educational institution	UK osteopaths *N* = 8 M = 4 F = 4	Constructivist grounded theory	Osteopaths viewed BPS model as essential in navigating a person’s experience of pain, however, the integration of the BPS model into clinical practice is fraught with obstacles.
Formica et al., (2018) [17] (Italy)	In-depth semi-structured interviews (individual)	Controlled interview setting	Italian osteopaths *N* = 11 M = 9 F = 2	Grounded theory	Italian osteopaths displayed a greater orientation towards the biomedical dimension of chronic pain
Delion & Draper-Rodi (2018) [32] (UK)	Semi-structured interviews (individual)	Teaching centre (university)	UK Final year osteopathic students *N* = 6 M = 3 F = 2	Constructivist grounded theory	Osteopathic students assessed for PS factors throughout the case history and tend to rely on instincts. However, barriers exist for managing PS factors when treating patients with NSLBP.
Draper-Rodi (2016) [33] (UK)	Mixed methods Study	Online e-learning program Educational institution	Quantitative strand *N* = 45 Qualitative strand *N* = 9	PABS ABS-mp Thematic analysis	A 6-week e-learning programme was feasible. The BPS approach was not structural enough.

*ABS-mp* Attitudes to Back Pain Scale for musculoskeletal practitioners, *BM* Biomedical, *BPS* Bio-Psycho-Social, *F* Female, *HC-PAIRS* Health Care Providers’ Pain and Impairment Relationship Scale, *M* Male, *N* Number of participants, *NSLBP* Non-Specific Low Back Pain, *PABS – PT* Pain Attitudes and Beliefs Scale for Physiotherapists, *PS* Psychosocial, *UK* United Kingdom

### Risk of bias in included studies

Two qualitative studies [32] met all of the 10 CASP appraisal items and one study [17, 31] met 8/10 criteria (Table 3).

**Table 3:** Risk of Bias of Included Studies Using Critical Appraisal Skills Program (CASP) Checklist

Study	Aims	Method	Research Design	Sampling	Data Collection	Reflexivity	Ethical Issues	Data Analysis	Findings	Value of Research
Abrosimoff & Rajendran (2020) [31]	Y	Y	Y	Y	Y	CT	Y	Y	CT	Y
Delion & Draper-Rodi (2018) [32]	Y	Y	Y	Y	Y	Y	Y	Y	Y	Y
Formica et al., (2018) [17]	Y	Y	Y	Y	Y	Y	Y	Y	Y	Y

*Y* Yes, *CT* Can’t Tell

Two quantitative studies [14, 15] scored a total of 3 out 4 points (75%) on the MMAT tool, and the mixed methods study [33] scored 9 out of 11 points (82%) (Table 4).

**Table 4:** Risk of Bias of Included Studies (CASP and MMAT scores)

Qualitative strand	Draper-Rodi (2016) [33]	MacDonald et al. (2018) [15]	Bar-Zaccay et al. (2018) [14]
1.1. Are the sources of qualitative data (archives, documents, informants, observations) relevant to address the research question(objective)?	Yes	N/A	N/A
1.2. Is the process for analysing qualitative data relevant to address the research question (objective)?	Yes	N/A	N/A
1.3. Is appropriate consideration given to how findings relate to the context, e.g., the setting, in which the data were collected?	Yes	N/A	N/A
1.4. Is appropriate consideration given to how findings relate to researchers’ influence, e.g., through their interactions with participants?	Yes	N/A	N/A
**Quantitative strand**			
4.1. Is the sampling strategy relevant to address the quantitative research question (quantitative aspect of the mixed methods question)? E.g., consider whether (a) the source of sample is relevant to the population under study; (b) when appropriate, there is a standard procedure for sampling, and the sample size is justified (using power calculation for instance).	Partially yes (no power calculation)	Yes	Yes
4.2. Is the sample representative of the population understudy? E.g., consider whether (a) inclusion and exclusion criteria are explained; and (b) reasons why certain eligible individuals chose not to participate are explained.	Yes	Yes	Yes
4.3. Are measurements appropriate (clear origin, or validity known, or standard instrument)? E.g., consider whether (a) the variables are clearly defined and accurately measured; (b) measurements are justified and appropriate for answering the research question; and (c) the measurements reflect what they are supposed to measure.	Yes	Yes	Yes
4.4. Is there an acceptable response rate (60% or above)? The response rate is not pertinent for case series and case report. E.g., there is no expectation that a case series would include all patients in a similar situation.	No (response rate – 8%)	No	No
**Mixed methods**			
5.1. Is the mixed methods research design relevant to address the qualitative and quantitative research questions (or objectives), or thequalitative and quantitative aspects of the mixed methods question (or objective)?	Yes	N/A	N/A
5.2. Is the integration of qualitative and quantitative data (or results*) relevant to address the research question (objective)?	Yes	N/A	N/A
5.3. Is appropriate consideration given to the limitations associated with this integration, e.g., the divergence of qualitative and quantitativedata (or results*) in a triangulation design?	Yes	N/A	N/A
**Total score**	9/11 (82%)	3/4 (75%)	3/4 (75%)

### Summary of advanced meta-integration synthesis

Two key concepts (current practice and embracing a BPS approach) were generated using advanced meta-integration synthesis. The key themes, subthemes and variables that were part of the meta-integration are summarized in Table 5. In the following section, direct participant quotes are displayed in “Italics” and author derived subthemes are displayed in ‘Roman’.

**Table 5:** Advanced meta-integration: synthesis of quantitative, qualitative, and mixed methods data

Concept	Quantitative (variable)	Qualitative (sub-theme)	Supporting quotes from included studies	MM-Quan (Variable)	MM-Qual (sub-theme)	Integration Concepts (with Themes and subthemes contributing)
**Current Practice (Attitude/Belief towards BPS)**	PABS.PT	Strong biomedical belief Lack of knowledge about tools to measure PS factors Fixed osteopathic belief (structural outlook) Training covered MSK pain only BPS is a vague concept BPS lacks definition	*“And I felt my training was very much like that [mechanically focused] …*. I *can’t say we weren’t taught these things [BPS model]”* *“I mean, I suppose I’m a bit of a structural osteopath in that I will always look for, I hate to say it, the ‘tissue-causing symptoms’”* *“I try always to identify the ‘structurality of the things’ and verify if there is something related to biological nature of pain (*e.g. *arthrosis) in that patient”* *“My osteopathic education was based on biomechanical-tissue model, that is my reference model, even because it represents what I know better and what makes me more confident and assured”* *“I believe that PS factors play a major role into patient presentation of symptoms, especially the LBP but I’m not too happy about the definition of PS factors … the term is so broad, that I really find it a bit blurred, unspecific.”* *“I believe that osteopaths are aware of the existence and integration of the psycho components in chronic pain, but in many cases these factors are underestimated by them [and] we manage these problems according to our experience”*	PABS.PT	BPS was not structural enough	**Concept 1: Current practice - Rooted in BM model** ***Theme 1: Anchored in BM model*** Trained to deal with MSK pain Structural outlook ***Theme 2: BPS-non specific approach*** Vague and non-specific concept definition Underestimating PS factors
**Towards a BPS model of care**	HC-PAIRS	Pain education Pain neuroscience Patient empowerment Embrace BPS - Aligning with contemporary practice Patient empowerment Improve self efficacy Understanding patient perspective Listening to patient stories Providing reassurance BPS-added value Changed practice	*“so with the journey of gaining health … it is to empower people so that they can take charge and control of their bodies and their health and their life”* *“It seems to be absolutely everywhere at the moment. It seems to be the way the NHS is going in this country, the way physios are going in this country so I think it’s something we need to embrace - that we need to be very aware of”* *“And also trying to move away from, you know, ‘Once your right SIJ is going to move well, you are going to feel much better’, sort of thing, having a, sort of, more context, more talk around their context, rather than just their body’”* *“The skill to reassure and keep patients calm is very important, and represents also a starting point in the management of chronic patients, especially if they are anxious or depressed”* *“We osteopaths are very different from the allopathic doctor; we establish with the patients a more superior verbal relationship”* *“We have a verbal and nonverbal conversation with the patients, especially through the touch and correct use of the words. The communication and the words have to be weighed and carefully evaluated”* *“I think that the therapeutic relationship is fundamental when I approach chronic patients. In any case, there are a lot of jobs to do, especially while searching to share outcomes with patients”* *“Listening, querying, questioning patients- in a way I’m questioning their beliefs, their thoughts in a way that they may think actually, ‘why am I doing that?’ I then offer them different approaches”* *“I think it could affect it in terms of their pain perception, so the pain processing, so where they interfere with kind of sensitisation, or altering descending inhibition within the central nervous system, or really focusing on pain, which can change their experience of it”* *“The patient’s active role is essential, because they are the main actors of this therapeutic relationship. I can help them with my treatments, but they are living in pain, and it is a partnership that we have”* *“I think one of the biggest skills is being able to sync with the patient regardless of who they are and how they are and just work it out together”*		Knowledge about BPS was a transformative experience	**Concept 2: Towards a BPS model of care** ***Theme 3: Embrace the BPS approach*** Futuristic model Foundational knowledge – pain and neuroscience ***Theme 4: Therapeutic alliance*** patient empowerment improve self-efficacy understanding patient perspective ***Theme 5: Evolution in practice*** Communication as a key role BPS-added value Changed practice

*BM* Biomedical, *BPS* Bio-Psycho-Social, *HC-PAIRS* Health Care Providers’ Pain and Impairment Relationship Scale, *MM* Mixed Methods, *NSLBP* Non-Specific Low Back Pain, *PABS* PT - Pain Attitudes and Beliefs Scale for Physiotherapists, *PS* Psychosocial, *Qual* Qualitative, Quan – Quantitative

### Usage of the BPS approach in current osteopathic practice

#### Current practice

**Fig. 2:**
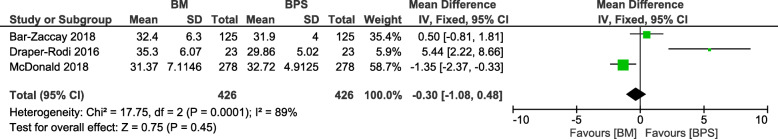
Meta-analysis of BM vs BPS approach adopted by osteopaths as indicated by PABS.PT scores

The quantitative variables measured were the attitudes and beliefs of osteopaths using the Attitudes to Back Pain Scale in musculoskeletal practitioners, Pain Attitudes and Beliefs Scale for Physiotherapists and Health Care Providers Pain and Impairment Relationship scale. Three studies [14, 15, 33] had utilised the Pain Attitudes and Beliefs Scale for Physiotherapists enabling pooling of data that indicated that osteopaths may prefer a biomedical approach (though not statistically significant) than the BPS approach in their clinical practice, refer Fig. 2.

These findings together with qualitative data indicate that current osteopathic practice is anchored mainly in the biomedical model. Most osteopaths perceived that they were “*trained to deal with musculoskeletal pain*” and therefore had “*more confidence in dealing with musculoskeletal*” aspects of a patient’s presentation than the non-musculoskeletal aspects [17]. This led osteopaths to focus their treatment on biomedical factors rather than broader BPS factors. Some osteopaths explained this as ‘having a structural outlook’ to their treatment approach and efforts were made to “*identify the tissue(s) causing symptoms*” [31, 34]. Hence, a ‘structural osteopath’ ruling out ‘structural issues’ was misconceived as utilizing a BPS approach. This resulted in structural osteopaths’ believing that they were utilizing some aspects of the BPS model and adopting a holistic approach to their treatment. However, this “*holistic approach”* was still rooted in biomechanics rather than including psychosocial factors [14, 34]. Osteopaths also perceived the BPS model as a ‘vague concept’ with a ‘non-specific approach’ as it “*did not sufficiently focus on structure*” [32]. Furthermore, ‘underestimating psychosocial factors’ as contributors to patient presentation, resulted in frustration as osteopaths were either “*unaware of objective tools*” (*or chose not to use it*) to measure psychosocial factors or had “*minimal access to referral pathway*s” to manage these patients [32]. This in turn led to confusion regarding the osteopath’s role/scope within the health system.

#### Embracing the BPS model

Osteopaths not only believed that the BPS model was “*everywhere now*” but also believed that it will be the ‘futuristic model’ of care [33]. This resulted in a perception that the osteopathic profession needed to ‘catch-up’ with the model as other health professionals such as “*physiotherapists are already using the model*”. They also believed that public health bodies (e.g. National Health Service) may prefer practitioners using a BPS approach and therefore “*osteopaths must embrace it*” [33]. Understanding patient’s perspective enabled osteopaths to form a positive ‘patient practitioner’ relationship that was not only empowering the patients but could also “*improve their self-efficacy*” [31]. Osteopaths consistently reported that “*communication plays an important role*” in establishing a positive and empowering ‘patient-practitioner’ relationship [34]. In this context, good communication is comprised of three key attitudes/attributes (1) listening to the patient’s story (2) providing reassurance, and (3) mindful clinical conversations [17, 31, 34]. Therefore, osteopaths believed the BPS model expanded their current clinical practice. For example, instead of “*correcting*” or “*fixing*” the body through mobilisation, osteopaths “*explored different aspects in one’s life*” that have an impact on their symptoms and stop them getting better [34]. By having a better understanding what a patient’s symptoms mean for them, osteopaths felt that their “*practice had completely changed*” evolving towards more of a BPS approach [33].

### Summary of qualitative synthesis

Two concepts (barriers and enablers) were informed from qualitative only data [17, 31, 32] (refer Table 6). The level of confidence synthesised using GRADE-CERQual was ‘low” for both the concepts. Evidence profile, which includes summaries of the review findings, information on the judgments for each CERQual component underlying the overall CERQual assessment and the overall assessment with its explanation have been presented in Table 7. Further, a summary of qualitative findings (SoQF) have also been presented in Table 8.

**Table 6:** Qualitative Thematic Synthesis

Concept	QUAL – (Sub-themes)	Concepts (with Themes and subthemes contributing)	Supporting quotes from included studies
**Barriers**	Undertrained/underprepared Lack of clinical reasoning Threat to professional identity Intuition based approach to PS factors Lack of tools to measure PS factors Avoid/underdiagnose PS Factors Discordant with osteopathic beliefs Not within my professional scope Lack of resources Listen but still do bio Lack of contemporary BPS education	**Concept 3 - Barriers for implementing a BPS approach** ***Theme 7: Undertrained to apply BPS model*** Lack of contemporary BPS education Intuition based - lack of clinical reasoning Lack of resources ***Theme 8: Inability to diagnose*** Lack of tools Avoid/underdiagnose PS factors ***Theme 9: Threat to professional identity*** Discordant with osteopathic philosophy Not within scope Listen but still address biomechanical issues	*“And I felt my training was very much like that [mechanically focused] I can’t say we weren’t taught these things [BPS model]. We were exposed to them but I think almost too early in the course. So by the time you come to third/fourth year in clinic [exams], it’s all in the background, it’s all gone”* *“My undergraduate training paid little attention to this [BPS] model. I feel more comfortable to manage biomechanical and postural aspects of the patient’s pain. I think that BPS model is valid with respect to the chronic pain management, but I have no competence and knowledge to apply this model in my practice”* *“Osteopathy is removing barriers to function in the classical osteo-pathic sense … we’ve lost the way trying to be what people expect; respectable, acceptable, payable by the state we don’t put our foot down and stand for the principles of osteopathy”* *“I have 4 boxes which I tick one or more of these [pain mechanisms], of which I think is going on with that patient, and by this time I am past the psychosocial, I’m on to bio now”* *“I have a little knowledge of this [BPS] model. I have no competence to evaluate other patient’s context. Of course I think that such factors are important in the presentation but I do not have the confidence to manage these situations”* *“I leave the assessment for BPS to my own understanding and my own perception of the person as a whole; I don’t think I have any structured way of assessing for BPS factors.”* *“you could say the profession is in an identity crisis because we’re told we can be the practitioner we want to be … It’s very broad which makes me excited”* *“We have to take into account also the lack of training in pain management and communication inside the undergraduate curricula in Italy. In fact, some aspects are poorly covered and under explored”* *“I initially look for those body language cues and how they present themselves, then how they verbalise, what they are feeling in term of what it feels like to them”* *“I don’t particularly have a guide, I mean you do have screening tools, which are probably efficient, like STarT Back, which are effective, but I don’t use it”* *“the whole structure governs function thing, …, unfortunately that seems to be the one mantra that everyone knows and it’s probably the worst because it’s, it sets everything up to become dualist so that, you know, there’s no room for psychosocial stuff”* “*I’m really questioning myself about which tools a student or a qualified osteopath has to assess for bio-psychosocial symptoms?”*
**Enablers**	Ongoing education CBT/motivational Pain education/ interviewing/mindfulness Funding and EBP Adopting a blended approach CPD opportunities/workshops Implementing BPS-exemplars Self-awareness of clients Superior to GP Palpation skills	**Concept 4: Enabler for implementing a BPS approach** ***Theme 10: Acknowledging and managing PS factors*** Acknowledge PS factors Management strategies Self-awareness of clients ***Theme 11: Education/CPD*** Ongoing education Workshops e-intervention	*“Our job is to understand their reality, the patient reality, and find out how they come to that point”* *“If we are talking about stress, I might suggest mindfulness, if we are talking about depression, I will push my patient to go out with friends and I will tell the patient to do activities very good for the LBP, to try to engage the patient in the treatment with me in the room, but also engage the patient outside with a personal social life, aiming at doing what the patient likes”* *“[on my desk] ‘I’ve got a note which says, ‘tell me your story’”* *“Introduce them to pain education, educate them through kind of pain is not equal to tissue damage, and stuff like that, I think it’s a good way of managing it … talk about stress and its effect on the nervous system, kind of using analogy to make in a way this is easy to understand as possible”* *“So, for that patient, strongly nociceptive patient, I would probably offer hands-on because there might be some sort of nociceptive input from somewhere, but I would also provide some form of, CBT or motivational interviewing or something for these psychosocial factors to try to decrease the risk of developing chronic pain for that patient”* *“I use mindfulness techniques, box breathing techniques, advice on lifestyle, and advice on exercises, anything that is relevant, that can in-fluence the social side or the psychological side, that would then be beneficial, impact on the LBP”* *“I’m interested in the crossover between psychotherapy and body work, getting through the layers of the body … working with the mind through that hands-on approach”* *“[The course] has changed in some of the language maybe that I would use with patients and just re-emphasizing thought positives and maybe not using quite so much medicalised language”*

*BM* Biomedical, *BPS* Bio-Psycho-Social, *CBT* Cognitive Behavioural Therapy, *CPD* Continuous Professional Development, *EBP* Evidence Based Practice, *GP* General Physician, *LBP* Low Back Pain, *PS* Psychosocial, *UK* United Kingdom

**Table 7:** CERQual Evidence Profile

Summary of review Finding	Studies Contributing to the review finding	Methodological Limitations	Coherence	Adequacy	Relevance	CERQual assessment of confidence in the evidence	Explanation of CERQual assessment
Barriers for utilizing the BPS model in clinical practice: osteopaths working in Europe identified key barriers in utilising the BPS model that included lack of education and/or diagnostic tools. Some osteopaths perceived the BPS model as a threat to their professional identity.	(Abrosimoff & Rajendran, 2020; Delion & Draper-Rodi, 2018; Formica et al., 2018)	Minor concerns	No or very minor concerns	Moderate concerns (only threes studies offering thin data).	Moderate concerns (partial relevance as studies were done only in Europe and varied settings including regulation).	**Low confidence**	Three studies with no methodological limitations, no concerns about coherence, limited, thin data from 2 countries, moderate concerns about adequacy and relevance.
Enablers for utilizing BPS model in clinical practice: factors that may enable/facilitate the use of BPS model by osteopaths include acknowledging psychosocial factors, management strategies and continuous professional development courses.	(Abrosimoff & Rajendran, 2020; Delion & Draper-Rodi, 2018)	Minor concerns	Minor concerns (some concerns about fit between the data from primary studies and the review finding).	Moderate concerns (only two studies offering thin data).	Moderate concerns (partial relevance as studies were done only in Europe and varied settings including regulation).	**Low confidence**	Two studies with minor methodological limitations, minor concern about coherence, limited, thin data from 2 countries, moderate concerns about adequacy and relevance.

**Table 8:** CERQual Summary of Qualitative Findings

Summary of review finding	Studies contributing to the review finding	CERQual assessment of confidence in the evidence	Explanation of CERQual assessment
Barriers for utilizing the BPS model in clinical practice: osteopaths working in Europe identified key barriers in utilizing the BPS model that included lack of education and/or diagnostic tools. Some osteopaths perceived the BPS model as a threat to their professional identity.	(Abrosimoff & Rajendran, 2020; Delion & Draper-Rodi, 2018; Formica et al., 2018)	**Low confidence**	Three studies with minor methodological limitations, no concerns about coherence, limited, thin data from 2 countries, moderate concerns about adequacy and relevance.
Enablers for utilizing the BPS model in clinical practice: European osteopaths identified various factors that may enable/facilitate the use of BPS model in clinical practice including acknowledging PS factors, management strategies and CPD courses	(Abrosimoff & Rajendran, 2020; Delion & Draper-Rodi, 2018)	**Low confidence**	Two studies with minor methodological limitations, minor concern about coherence, limited, thin data from 2 countries, moderate concerns about adequacy and relevance.

### Factors that prevent or enable osteopaths to implement a BPS approach

#### Barriers for implementing a BPS approach

Osteopaths identified several factors that were barriers (low confidence) in implementing a BPS approach. A key barrier was that osteopaths felt that their “*undergraduate education was based on the biomechanical-tissue model*” and “*paid little attention to the BPS model*” [31, 32]. Hence, these osteopaths were more confident in managing the biomechanical and postural aspects of the patient’s pain than dealing with patient’s context and situation [17, 31, 34]. Lack of contemporary BPS education and resources left osteopaths to adopt an ‘intuition-based approach’ with little or no clinical reasoning to determine when to/not to apply the BPS model. While, some osteopaths were not aware of objective tools to “*assess bio-psychosocial symptoms*”, others were aware of objective tools to measure psychosocial factors (e.g. STarT Back questionnaire) but chose “*not to use it*”. This led osteopaths to either ‘avoid or underdiagnose’ the contribution that psychosocial factors may have in their patient’s symptoms [32]. This combined with evidence-based clinical guideline recommendations led osteopaths to perceive that the “*BPS approach devalues what they do*”, and therefore ‘threatens their professional identity’ [31]. This need for professional identity perpetuated the use of a structural/biomechanical approach as they felt “*comfortable to manage biomechanical and postural aspects of the patient’s pain*”. Hence, managing the psychosocial aspect of a patient’s presentation was believed to be ‘outside the scope of osteopathic practice’ [17, 32].

## 4: Enablers for implementing a BPS approach

Osteopaths identified several factors that enabled (low confidence) them to implement a BPS approach in their clinical practice. A key factor was ‘acknowledging the relevance of assessing psychosocial factors’ in a patient presentation [32, 34]. Giving consideration to psychosocial factors enabled the osteopaths to utilize a broader approach that was more patient centred. For example, educating patients about pain and making them understand that “*pain is not equal to tissue damage*” was considered a good ‘management strategy’. Other strategies such as cognitive behavioural therapy (CBT), mindfulness and motivational interviewing may also facilitate osteopaths to make their clients become more ‘self-aware’ of their condition, with an eye to trying “*not to create dependency*” on passive therapies [31, 32, 34]. Osteopaths reported that learning opportunities such as via continued professional development (CPD), helped them to integrate the BPS model in their practice. They also reported that other ‘ongoing education and/or workshops’ would be vital to support osteopaths in implementing a BPS approach [33]. While some osteopaths acknowledged the importance of including the BPS model as part of their undergraduate training, other osteopaths preferred to have this type of training be conducted at the postgraduate or professional development level [33, 34].

## Discussion

### Summary

This study aimed to understand the enablers and challenges towards implementing BPS approach in osteopathic practice. The findings from this review suggests that, despite various guidelines recommending the use of the BPS model, current osteopathic practice predominantly occurs within the biomedical model of care. Findings suggests that osteopaths may be aware of the theoretical underpinnings of the BPS model and identify the need to embrace it. However, barriers such as lack of contemporary education about the BPS model; structural approach to diagnosis and treatment; lack of and/or choosing not to use objective tools to measure psychosocial factors; and threat to professional identity may prevent them from using the BPS approach. On the other hand, ongoing education and/or workshops may facilitate osteopaths to implement the BPS approach.

### Comparison with existing literature

To our knowledge, this is the first systematic exploration of current use of the BPS model by osteopaths in clinical practice. Our findings suggest that most osteopaths consider patients’ psychosocial concerns might have clinical importance and recognised the need to manage these factors as they have the potential to influence recovery. However, despite having a theoretical understanding of the BPS model, some osteopaths struggled to find strategies to incorporate it into clinical practice. This transitional challenge from theory to practice is consistent with previous findings [31, 35] and in other similar musculoskeletal professions other professions such as physiotherapy [21, 36].

Our findings suggest that current osteopathic clinical practice may largely be situated in the biomechanical paradigm, [14, 19]. These structurally dominated concepts may highlight the importance that osteopaths ascribe to biomechanical and anatomical features they felt they could measure [37]. Osteopaths relied on their ‘instincts’ following observation of non-specific patient behaviours, such as posture to assess psychosocial influences. Osteopaths seemed to either be unaware of tools for assessing psychosocial factors or in some cases avoided using them. This practice may result in under/misidentification of psychosocial factors and therefore influence management strategies. As a growing body of evidence indicates that psychosocial factors contribute to a patient’s presentation and recovery, it may be imperative that they are addressed effectively and appropriately in the context of the individual patient [5, 38] through early identification and assessment in the clinical setting [39].

A unique finding of our review was that some osteopaths believed the BPS model has become the dominant approach in osteopathic practice in future and therefore thought it may be a good strategy to embrace it. These osteopaths believed that working within the BPS model may enable them to use more appropriate management strategies such as pain education and psychological approaches. For example, some osteopaths identified that understanding various psychological approaches such as CBT and mindfulness may help them to understand the importance of psychological factors and the need to address these factors effectively in their clinical practice. The understanding about various strategies may be crucial as a Cochrane review has clearly highlighted that patients with chronic low back pain receiving multidisciplinary biopsychosocial rehabilitation programs are likely to experience less pain and disability than those receiving usual care or a physical treatment [40]. It is important to note that the psychological care was provided by psychologists in the Cochrane review. However, these findings may be considered as emerging evidence signalling a paradigm shift from a ‘biomedical’ model towards a ‘person-centred’ management in osteopathy.

Our review found ‘low confidence’ that various barriers may prevent osteopaths from implementing a BPS model of care. A key barrier was a perception that the BPS model is a threat to osteopaths’ ‘professional identity’. Some osteopaths [31] considered that osteopathy by its very nature (and therefore their current practice) was already holistic and therefore consistent with the BPS approach,. However, the ‘holistic’ models were ‘biomechanical’ in nature and grounded in a biomedical paradigm which may question the understanding some osteopaths may have regarding ‘patient centredness’ [41].

Our review found ‘low confidence’ to strategies that may facilitate the use of the BPS model care by osteopaths. Consistent with previous findings, osteopaths felt they had received inadequate undergraduate and/or postgraduate training to effectively explore, assess and manage psychosocial factors [31, 32]. Therefore ongoing focused education and/or workshops may facilitate incorporating the BPS model in clinical practice, which is in line with previous findings [17, 42]. Preliminary evidence suggests that an 8-h e-learning program shifted the osteopaths’ attitudes towards a more BPS view of back pain [33]. However, it has to be noted that such educational/CPD strategies can be expensive and time consuming, which can deter practitioners from attending such courses [42]. There has been an increasing call to incorporate the assessment of psychosocial factors as part of graduate level osteopathic programs [31, 32]. However, successfully incorporating the BPS model into graduate curriculum, may require it to be in place at undergraduate level. Furthermore, it may be important for undergraduate/new graduates to observe patient centred practice/behaviours modelled in professional practice and clinical placements.

#### Strengths and limitations

This review had several strengths. We used a comprehensive search strategy that included white and grey literature search to maximise chances of locating all relevant studies representing the phenomena of interest. We published our protocol in advance to promote transparency and we did not deviate from the published protocol [24]. Two authors independently conducted each major review processes (study search, study selection, data synthesis and analysis) to reduce bias and error. We used advanced meta-integration synthesis to enable combination of quantitative and qualitative data that may increase the confidence in our findings.

However, a number of limitations are worth considering. Firstly, a key limitation was that our findings are based on a low number (six) of included studies [14, 15, 17, 31–33]. Our findings are of ‘low confidence’ as we have moderate concerns about their relevance given all studies were conducted in Europe (despite including various settings). Further, most qualitative studies were done in only one institution (UCO) which may limit the generalisability of our findings. We also have moderate concerns about the adequacy of the findings as only three qualitative studies contributed rich data [17, 31, 32]. We included one study [32] of osteopathic students (consistent with the inclusion criteria). The themes that emerged from this study were consistent with studies of qualified osteopaths and served to strengthen findings rather than altering interpretations. Another limitation is that we only included English language studies leading to language bias. One member of the current review team (OT) was part of authorship of two studies included in the review, though they did not have any role in the data extraction and critical appraisal process.

#### Implication for practice

Though our findings provide evidence of an emerging acknowledgement of the importance of BPS model of care, the biomedical model seems to still dominate osteopathic clinical practice. Hence, osteopaths may miss opportunities to enhance health of their patients by not being able to identify and manage psychosocial factors. A paradigm shift therefore may be necessary as clinical practice guidelines [11, 12] commonly recommend assessment and treatment of physical and psychosocial factors in an appropriate, effective and meaningful way. An important finding from this review was that there seems to be a lack of understating of psychosocial factors and their assessment by osteopaths which was in turn associated this with lack of education at an undergraduate level. Importantly, osteopaths who participated in online education and/or had exposure to the BPS model felt that they had access to more treatment strategies [33]. Hence, it may be imperative for osteopathic education of psychosocial assessment to be reviewed and strengthened.

#### Implication for research

There is dearth of osteopathic research related BPS aspects of practice, particularly outside of the UK. Therefore, it may be of important priority for research on BPS model of care in osteopathic practice to be undertaken in other countries. This may help us to identify unique factors such as culture, patient demographics, health policies, etc. that may influence osteopaths to use BPS model in their clinical practice. Further research is required to tease out the challenges in translating theoretical knowledge about the BPS model to clinical practice. Research investigating the extent of coverage of BPS model in undergraduate curriculum across countries may be timely. Such a research may also identify the most effective ways to teach students about psychosocial assessment and management in osteopathic clinical practice. In terms of post-graduate training or CPD activities, future research may investigate the best ways to deliver these educational packages.

## Conclusion

Our review findings suggest that osteopaths may be aware of the theoretical underpinnings of the BPS model and identified the need to embrace it. However, some key barriers related to education about the BPS model, objective tools to measure psychosocial factors and professional identity may prevent osteopaths from using it their clinical practice. Therefore, ongoing education and/or workshops may be necessary to enable osteopaths to implement a BPS approach. Our findings are based on small number of studies pointing to the limited evidence on this topic and the need for more robust studies in this area.

## Data Availability

The datasets used and/or analysed during the current study available from the corresponding author on reasonable request.

## Abbreviations

ABS-mp: Attitudes to Back Pain Scale for musculoskeletal practitioners
BM: Biomedical
BPS: Bio-Psycho-Social
CASP: Critical Appraisal Skills Program
HC-PAIRS: Health Care Providers’ Pain and Impairment Relationship Scale
MMAT: Mixed Methods Appraisal Tool
NSLBP: Non-Specific Low Back Pain
PABS: PT Pain Attitudes and Beliefs Scale for Physiotherapists
PRISMA: Preferred Reporting Items for Systematic reviews and Meta-Analysis
PROSPERO: Prospective Register of Systematic Reviews
PS: Psychosocial
UK: United Kingdom
